# Bacteria–zinc co-localization implicates enhanced synthesis of cysteine-rich peptides in zinc detoxification when *Brassica juncea* is inoculated with *Rhizobium leguminosarum*

**DOI:** 10.1111/nph.13588

**Published:** 2015-08-11

**Authors:** Gbotemi A Adediran, Bryne T Ngwenya, J Frederick W Mosselmans, Kate V Heal

**Affiliations:** 1 School of GeoSciences, The University of EdinburghEdinburgh, EH9 3JW, UK; 2 Diamond Light SourceHarwell Science and Innovation Campus, Didcot, OX11 0DE, UK

**Keywords:** bacterial localization, *Brassica juncea*, cysteine, plant growth promoting bacteria, X-ray absorption spectroscopy, zinc (Zn) accumulation, Zn detoxification, Zn speciation

## Abstract

- Some plant growth promoting bacteria (PGPB) are enigmatic in enhancing plant growth in the face of increased metal accumulation in plants. Since most PGPB colonize the plant root epidermis, we hypothesized that PGPB confer tolerance to metals through changes in speciation at the root epidermis.
- We employed a novel combination of fluorophore-based confocal laser scanning microscopic imaging and synchrotron based microscopic X-ray fluorescence mapping with X-ray absorption spectroscopy to characterize bacterial localization, zinc (Zn) distribution and speciation in the roots of *Brassica juncea* grown in Zn contaminated media (400 mg kg^−1^ Zn) with the endophytic *Pseudomonas brassicacearum* and rhizospheric *Rhizobium leguminosarum*.
- PGPB enhanced epidermal Zn sequestration relative to PGBP-free controls while the extent of endophytic accumulation depended on the colonization mode of each PGBP. Increased root accumulation of Zn and increased tolerance to Zn was associated predominantly with *R. leguminosarum* and was likely due to the coordination of Zn with cysteine-rich peptides in the root endodermis, suggesting enhanced synthesis of phytochelatins or glutathione.
- Our mechanistic model of enhanced Zn accumulation and detoxification in plants inoculated with *R. leguminosarum* has particular relevance to PGPB enhanced phytoremediation of soils contaminated through mining and oxidation of sulphur-bearing Zn minerals or engineered nanomaterials such as ZnS.

## Introduction

Zinc (Zn) is a constituent of many enzymes and proteins that sustain life, and is thus an essential trace metal for optimum growth and development in plants (Broadley *et al*., 2007). However, it is also phytotoxic at elevated concentrations, with plants exhibiting stunted root and leaf chlorosis (Frérot *et al*., 2005). Zinc toxicity in plants is one of the most extensive microelement phytotoxicities (Chaney, 1993). Plant leaf accumulation of < 100 mg kg^−1^ Zn dry weight (DW) generally hinders normal plant growth, although leaf chlorosis is not normally apparent until the zinc concentration exceeds 300 mg kg^−1^ leaf DW (Chaney, 1993; Broadley *et al*., 2007).

Different plant species tolerate zinc to different levels (Ebbs & Kochian, 1997; Shen *et al*., 1997) and different mechanisms of tolerance to elevated concentrations of zinc have been suggested (Clemens, 2001). For example, the binding of zinc to more tolerant organelles such as root cell walls and cell membranes reduces the zinc concentration in other zinc sensitive organelles in the root endodermis (Krzesłowska, 2011; Lang & Wernitznig, 2011). Moreover, the vacuole has been shown to be the main plant organelle responsible for storing toxic compounds in plant cells (Clemens, 2006). Rapid efflux of zinc from the cytosol into the vacuoles and subsequent vacuolar sequestration of excess zinc has also been suggested as a possible mechanism of zinc tolerance in plants (Kobae *et al*., 2004). Apart from subcellular compartmentalization of zinc, the reduction of zinc bioavailability in plant cells through Zn complexation by phytochelatins and metallothioneins has also been suggested as a key mechanism behind plant tolerance to elevated Zn concentrations (Cobbett & Goldsbrough, 2002; Hossain *et al*., 2012).

Besides these plant-specific mechanisms, inoculation with plant growth promoting bacteria (PGPB) has been shown to confer tolerance to plants exposed to toxic concentrations of zinc (Burd *et al*., 1998; Wani *et al*., 2007). Some of these PGPB also increase zinc bioaccumulation while enhancing plant growth, a process that is being explored for detoxifying zinc contaminated environments (Li *et al*., 2007; Long *et al*., 2013). Most of these PGPB colonize plant roots and are closely associated with the root epidermis, the external plant regions including the root rhizosphere, rhizosplane and part of the root cortex (Compant *et al*., 2010; Dimkpa *et al*., 2014). Hence, it can be inferred that an important mechanism through which PGPB confer tolerance to metals is through enhanced metal compartmentalization at the root epidermis. However, to our knowledge, this has never been demonstrated. Similarly, it is unclear whether PGPB attenuate toxicity through bacteria induced changes in zinc chelation at the root epidermis.

In this study bacterial localization, zinc distribution and speciation in the roots of *Brassica juncea* grown in zinc contaminated media with and without *Pseudomonas brassicacearum, Rhizobium leguminosarum* and a combination of the two bacteria was characterized using, for the first time, a combination of fluorophore based confocal laser scanning microscopic imaging and synchrotron based microscopic X-ray fluorescence mapping and X-ray absorption spectroscopy. The main aim was to establish whether the spatial distribution of zinc in the root was related to that of bacteria and hence whether there were differences in root zinc speciation in relation to the distribution of bacteria. We focused on the early stages of growth, having demonstrated in our previous study (Adediran *et al*., 2015) that the effects of these PGPB were manifested in the first 2 weeks, allowing us to focus on the role of the different bacteria in metal speciation changes in these early stages.

For the microscopic analysis described in this study, the plant root was investigated as two main cellular regions: the epidermis, the area of plant root extending from the cell wall to *c*. 5.0 mm into the cortex; and the endodermis, the inner cell area within the membranes of the endodermis, including the vacuole and the vascular bundles (Di Laurenzio *et al*., 1996).

It was hypothesized that:

- *P. brassicacearum* and *R. leguminosarum* will colonize the endodermis and epidermis of the root, respectively, since the former is an endophytic bacterial strain of Brassica roots (Achouak *et al*., 2000) and the latter is a rhizospheric bacterial strain isolated from the root of a clover plant (Reeve *et al*., 2010).
- The PGPB will co-localize with zinc at the root epidermis and enhance epidermal zinc sequestration since root exudates (rhizodeposits) that often attract high bacterial populations are mostly at the root epidermis (el Zahar Haichar *et al*., 2008; Compant *et al*., 2010).
- The PGPB will enhance the storage of less toxic zinc species in the endodermis of *B. juncea* root where metal sensitive organelles are located compared with the un-inoculated plants.

## Materials and Methods

*Brassica juncea* L. Czern., a well-known accumulator of zinc (Sridhar *et al*., 2005; Adediran *et al*., 2015), was studied. *Pseudomonas brassicacearum* subsp.* brassicacearum* (strain DBK11) isolated from the root of a Brassica plant (Leibniz Institute DSMZ, Germany) and *Rhizobium leguminosarum* bv.* trifolii* (strain WSM1325) isolated from the nodules of a clover plant (School of Biological Sciences, University of Edinburgh, UK) were used as plant growth promoting bacteria. These strains were selected for their ability to colonize plant roots, promote plant growth and enhance zinc accumulation in *B. juncea* plant roots and shoots (Adediran *et al*., 2015).

The growth medium used for the seed germination and seedling growth was sterilized Tork advanced wiper 420 centerfeed roll M2 system paper placed in sterile Petri dishes. It is similar to the roll towel test (Ma *et al*., 2011) and the plant growth promotion assay on filter paper developed by Glick *et al*. (1995) and modified by Belimov *et al*. (2001) which has been used to assess root growth promotion in bacteria inoculated on *B. juncea* plants exposed to cadmium toxicity (Belimov *et al*., 2005) and in *Orychophragmus violaceus plants exposed to* zinc (He *et al*., 2010). The medium sustains *B. juncea* plant growth for 14 d after seed planting without nutrient supplements.

Zinc sulphate solution was used as the source of zinc contamination for this study since this is the dominant form of zinc contamination in oxic soils (Mulligan *et al*., 2001; Broadley *et al*., 2007). The experiment was conducted under a sterile laminar flow cabinet at 25°C with artificial lighting used to simulate 12 h : 12 h, day : night photoperiods.

### Experimental treatments

A completely randomized design of eight treatments, each comprising eight replicate Petri dishes, was set up in a laminar flow hood. Plant growth parameters were assessed from six replicates, while the remaining two replicates were used for XAS analysis and confocal laser scanning microscopy (CLSM). For each of the experimental replicates, 5 g of sterile wiper paper (folded as 2.5 g into the base and cover of the Petri dishes, respectively) was placed into sterile Petri dishes. The wiper paper filled Petri dishes were then exposed to UV light for 30 min to remove any bacterial contamination. For the zinc contamination treatments, a 400 mg l^−1^ zinc sulphate solution was prepared with sterile deionized water, filtered through a 0.22 μm syringe filter to remove any bacteria and 5 ml was applied to 5 g of the wiper so that all the solution was absorbed by the wiper to give an application rate of 400 mg kg^−1^ Zn. Since the pH of the zinc contaminated wiper determined in deionized water was 6.4–7.2 the possibility of plant toxicity in the experiment being caused by acidity was low and the pH of the zinc solution was therefore not adjusted. Speciation calculations using Visual Minteq showed that 75% of the added zinc was in the form of Zn^2+^_(aq)_ with the rest as aqueous ZnSO_4_ ion pairs, which have a low stability constant (see Discussion). Five millilitres of sterile deionized water was added to the controls without zinc to ensure all treatments started with the same moisture content.

For treatments that required bacteria inoculation, seeds of *B. juncea* were surface sterilized with 0.05 M sodium hypochlorite, washed three times in sterile deionized water and soaked under aseptic conditions in a bacteria-sterile water suspension of 7.5 × 10^8^ CFU ml^−1^ at 30°C for 3 h. Seeds for treatments without bacteria inoculations were soaked in sterile deionized water for the same duration under the same experimental conditions. For each replicate, 12 seeds were placed in a 4 × 3 grid pattern in the wiper filled Petri dishes. The Petri dishes were kept moist with sterile deionized water throughout the experiment.

### Seedling growth assessment

Fresh root and shoot lengths were measured and the total dry biomass of seedlings in each plate was determined by drying the biomass at 70°C to a constant weight and weighing at 14 d after seed planting. Zinc tolerance indexes, which estimate the ability to resist stress, were calculated separately for the roots, shoots and the whole seedling as: (*LZn*/*Lo*) × 100, where *LZn* is the shoot/root length and total dry biomass in replicates contaminated with zinc and *Lo* is the equivalent value in uncontaminated replicates for each treatments (Shi & Cai, 2010). The means of the zinc tolerance indexes from the six replicates were then calculated for the treatments.

### Preparation of plant roots for microscopic imaging

In order to ensure the acquisition of high resolution images of bacteria and zinc in the internal cell structure of plant roots, thin sections of 35 μm thickness of root biomass were acquired using a microtome under cryogenic conditions. Comparing sections with the same thickness was critical to demonstrating bacteria–zinc co-localization using X-ray absorption spectroscopy so that differences in the count rates due to high concentrations associated with bacteria cells could not be confounded with those due to larger thickness variations in samples. Roots were carefully harvested and immediately cryo-fixed in optimum cutting temperature compound (OCT) at −80°C to preserve the structural integrity of the sample and to ensure ease of handling during sectioning (Knapp *et al*., 2012). Cryo-embedded samples were cut into uniform sections with a Leica CM1900 cryostat at a temperature of −35 to −25°C. Sections for microscopic imaging were collected on standard microbiology glass slides while sections to be analysed by XAS were collected on sterile Kapton® tape placed on glass slides and secured with adhesive tape. The adopted biomass sectioning method ensured minimum disturbance of bacteria/metal association and distribution. Sterility was maintained throughout the sample preparation process.

### Confocal laser scanning microscopy (CLSM)

Sectioned root samples on glass slides were stained with a nucleic acid stain SYTO®9 and a metallic ion stain RhodZin™-3 for bacteria and zincimaging, respectively. The green fluorescent SYTO9 stain is capable of penetrating all types of bacterial cytoplasmic membrane and it is used to assess the total bacteria population (Berney *et al*., 2007). RhodZin-3 is an orange fluorescent indicator for Zn^2+^ ions (Sabnis, 2010) capable of penetrating cell membranes and has been used previously for investigating the physiological consequences of Zn^2+^ in living cells (Kikuchi *et al*., 2004). Both stains do not contain any metal and both are water soluble at room temperature (Sabnis, 2010). The different maximum excitation/emission spectra of SYTO9 (484/498 nm; (Stocks, 2004) and RhodZin-3 (549/576 nm; (Sabnis, 2010) allows for simultaneous staining and imaging of zinc and bacterial cells in the plant biomass.

A mixed stain containing 0.83 μg ml^−1^ of SYTO9 and 5.0 μg ml^−1^ of Rhodzine-3 was prepared with sterile deionized water under aseptic conditions, and the plant samples were stained and incubated in the dark for 30 min before microscopic imaging. Stained slides were covered with 20 mm × 50 mm no. 1.5 cover slides and sealed with nail polish. Stained samples were imaged with a Leica SP5 confocal laser scanning microscope (CLSM). For all the samples imaged with the CLSM, at least 12 μm thick z-stack images were acquired at a 0.5 μm z-interval. This image acquisition method affords a step-wise study of sample cross-sections along the sample’s depth. Serially collected images were then reconstructed into 3-dimensional images using Image J software (Schneider *et al*., 2012), allowing us to distinguish colonization patterns of the two bacteria.

### Micro X-ray fluorescence spectroscopy (μ-XAS)

Sectioned plant samples collected on Kapton® tape were preserved in dry ice and transported to the Diamond Light Source, UK for μ-XRF mapping on the microfocus beamline I18. All samples were analysed within 3 d of harvesting from the zinc contaminated media. The cryo-fixing method used to preserve the samples has been reported to be effective for preserving biological samples for up to 4 wk (Knapp *et al*., 2012). Samples were mounted on the beam sample holder at an angle of 45° to the incident beam of 2.5 μm beam size. The beamline energy was calibrated using a Zn foil (9661 eV). Synchrotron micro X-ray fluorescence (μXRF) data for the samples were collected in fluorescence mode using a nine-element germanium solid state detector. The beam detector was at the same distance from the sample for the acquisition of the zinc maps for all treatments. The collected μXRF data were processed into images using PyMCA 4.4.1 (Solé *et al*., 2007). Points (six at the root epidermis and three at the root endodermis) displaying high zinc concentrations were selected from the μXRF images for microfocus X-ray absorption near edge structure (μXANES) analysis. Consecutive spectra from the same point were examined for possible beam damage and only the best spectra were used for μXANES analysis. Two samples from each of the treatments were analysed.

In order to determine the zinc speciation in the collected μXANES spectra for samples, Zn K-edge μXANES spectra were also collected under similar beam conditions for selected zinc standards – Zn oxalate, Zn phosphate, Zn histidine, Zn cysteine, Zn phytate, Zn polygalacturonate, Zn formate, Zn sulphate, Zn nitrate, Zn citrate, Zn acetate and Zn carbonate (see Supporting Information Table S1). Zinc cysteine was used as a model of the more complex thiol peptides glutathione and phytochelatin, which also contain cysteine-rich moieties and because previous studies have shown that cysteine and glutathione coordination is indistinguishable in XAS (Lu, 2013). Other reference compounds were chosen based on demonstrated coordination with zinc in plants and other zinc complexes that have been reported to be involved in zinc dynamics within the metal–bacteria–plant system studied (Kopittke *et al*., 2011; Adediran *et al*., 2015). Zinc standards were used in linear combination fitting (LCF) using a least-squares algorithm of the sample μXANES spectrum from 9645.3 to 9725.3 eV. The fractional contribution of each of the analysed standard compounds to the zinc spectrum was assumed to be directly proportional to the fraction of zinc present in that form in the plant root (Terzano *et al*., 2008). The goodness of the fit was estimated by calculating the residual *R* factor of the fit; *R *= Σ_i_ (experimental-fit)^2^/Σ_i_ (experimental)^2^. The sums (Σ) are over 103 data points as flattened mu (E). A lower *R* factor represents a better match between the fitted standard spectra and the sample spectrum (Terzano *et al*., 2008).

### Statistical analysis

Mean growth parameter values were calculated for every Petri dish yielding six values for each treatment (*n *=* *6). For the μ-XAS analysis, two plants were analysed per treatment (*n *=* *2), comprising one plant selected from the two replicated dishes. The six zinc spectra collected from the epidermis of each plant root and three from the endodermis of each plant root were merged per treatment and means of the percentage zinc compound compositions at the epidermis and endodermis of the two replicates per treatment were calculated. All treatment means were normally distributed and are of equal variance. One-way analysis of variance followed by Tukey’s HSD test (*P *<* *0.05) was used to identify significant differences between treatment means. All statistical analyses were conducted using Minitab 16 software (Minitab™ Inc., State College, PA, USA).

## Results

### Effect of PGPB on growth

**Figure 1:**
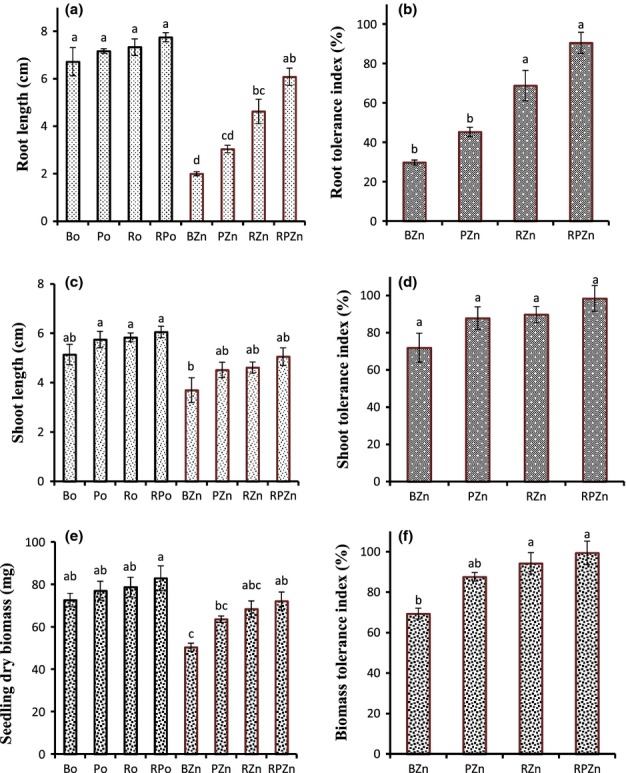
(a, c, e) Root length, shoot length and seedling biomass in un-inoculated *Brassica juncea* (B), *B. juncea* inoculated with *Pseudomonas brassicacearum* (P), *Rhizobium leguminosarum* (R) and combinations (RP) in uncontaminated treatments (o) and under zinc (Zn), and (b, d, f) root, shoot and seedling biomass tolerance indexes under Zn showing better root and biomass tolerance in treatment inoculated with *R. leguminosarum* and its combination with *P. brassicacearum*. Bars are means of root length, shoot length, seedlings biomass and % TI(s) from six Petri dishes each containing 12 seedlings. Error bars show standard errors. Different letters indicate significant (*P *< 0.05) differences in plant growth parameters between treatments.

Root, shoot and total seedling growth was assessed 14 d after planting and the percentage zinc tolerance indexes (TI) were calculated. In treatments without zinc contamination there were no significant differences in the root length between the bacterial inoculated treatments (Po, Ro and RPo) and the treatment without bacterial inoculation (Bo) (Fig.1a). However, under zinc contamination, root growth in the BZn treatment was stunted while there was better plant root growth in treatments inoculated with PGPB, especially in RZn and RPZn treatments. The mean root tolerance of the BZn plants to the 400 mg kg^−1^ Zn contamination was 30% (Fig.1b). Although inoculation with *P. brassicacearum* improves root tolerance to *c*. 45%, the effect was not statistically significant. In the presence of *R. leguminosarum* and of the two bacterial strains in combination, root tolerance to zinc contamination was significantly enhanced from 30% to 69% and 90%, respectively. There was no significant difference in the shoot length between treatments after the same 14-d period, apart from a shorter shoot length for the BZn treatment compared with the uncontaminated treatments with bacteria inoculation (Fig.1c). Shoot tolerance values were generally higher compared with root tolerances and there were no significant differences in the shoot tolerance to zinc between treatments with and without bacterial inoculation (Fig.1d). Roots are the main organ for zinc extraction and it is likely that at this stage of seedling growth, most of the zinc is mainly in the root.

For the seedling dry biomass determined in harvested roots and shoots at the end of the experiment, there was no significant difference in the uncontaminated treatments between bacterial inoculated treatments and the control (Bo) (Fig.1e). Under conditions of zinc contamination, biomass in the BZn treatment without bacterial inoculation was significantly lower compared with the uncontaminated (Bo). It is worth noting that seedling biomasses in the PZn, RZn and RPZn treatments were not significantly different from the biomass in the Bo treatment, an indication that seed inoculation with *R. leguminosarum* and its combination with *P. brassicacearum* helps the plant to recover full growth under zinc contamination. Moreover, although inoculation with *P. brassicacearum* improves the mean *B. juncea* seedling biomass zinc tolerance from 69.4% to 87.6%, the most significant effects were observed under inoculation with *R. leguminosarum* (RZn) and bacterial combinations (RPZn) with a TI of 94.3% and 99.4%, respectively (Fig.1f). A plant that completely resists metal toxicity and exhibits its growth potential under metal contamination has a metal tolerance index value of 100 ± 5%. It can therefore be inferred that inoculation of *B. juncea* seeds with a combination of *P. brassicacearum* and *R. leguminosarum* conferred complete tolerance (TI of 99.4%) to *B. juncea* seedlings established in 400 mg kg^−1^ Zn for 14 d.

The colonization of plant roots by bacteria and the ability of the bacteria to maintain a stable relationship with roots are important for effective plant growth promotion by bacteria (Lugtenberg & Dekkers, 1999; Compant *et al*., 2010). Bacteria inoculation and colonization of plant roots has also been observed to facilitate metal bioaccumulation (Adediran *et al*., 2015) with enhanced epidermal compartmentalization of metals suggested as a metal detoxification mechanism in plant roots (Krzesłowska, 2011; Lang & Wernitznig, 2011). In order to understand the possible bacterial enhancement of epidermal zinc sequestration as a mechanism behind the bacteria induced root tolerance to zinc contamination observed in this experiment, the localization of the bacteria strains and zinc in the seedling root was examined 14 d after planting.

### CLSM imaging of bacteria and zinc in plant roots

**Figure 2:**
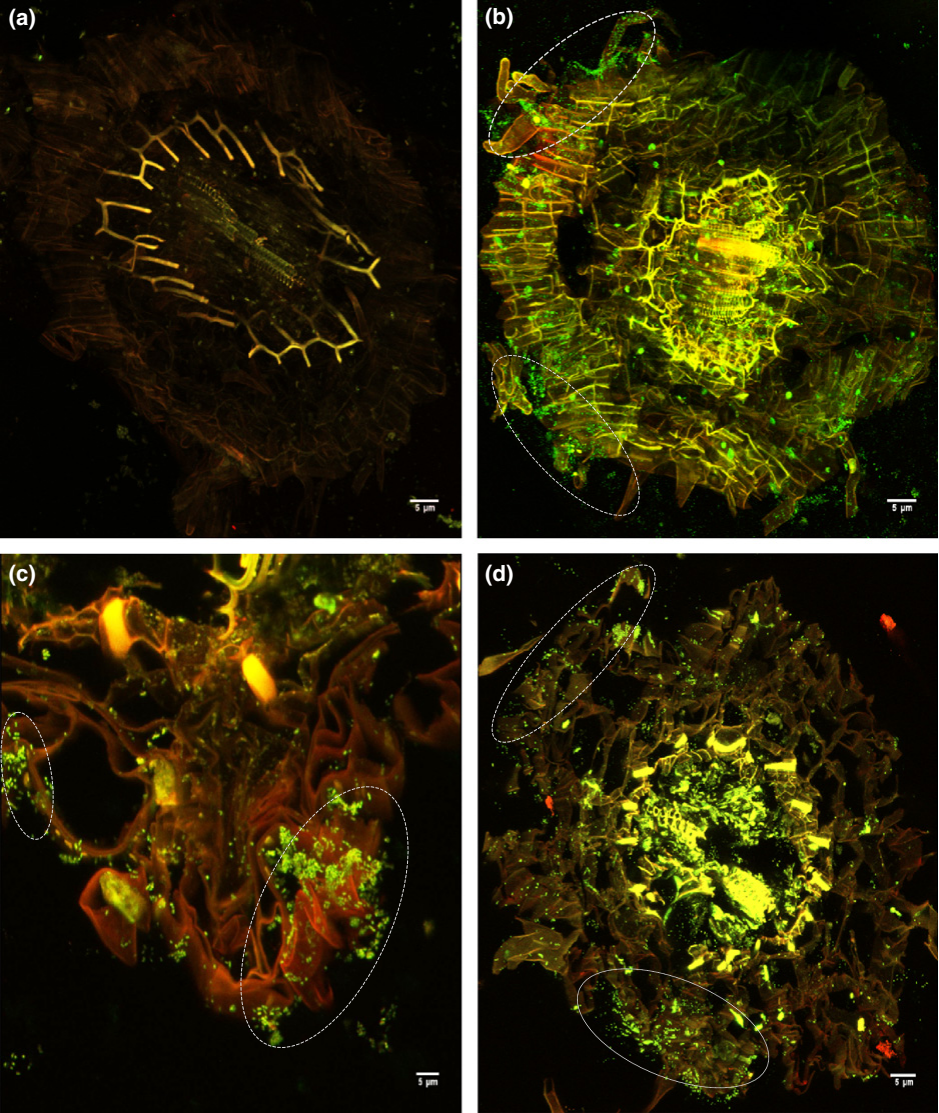
3-D reconstruction of confocal laser scanning microscopy (CLSM) images of (a) *Brassica juncea* root un-inoculated, (b) inoculated with *Pseudomonas brassicacearum*, (c) *Rhizobium leguminosarum* and (d) both bacterial strains, 14 d after exposure to 400 mg kg^−1^ zinc (Zn). Green fluorescent bodies are bacteria cells and the orange spots are areas of Zn localization. Note that the strains in the co-inoculation treatment (d) could not be distinguished since they both appear green. Areas of high bacteria localization along the root epidermis are circled. Figure shows absence of bacteria cells in the un-inoculated root.

**Figure 3:**
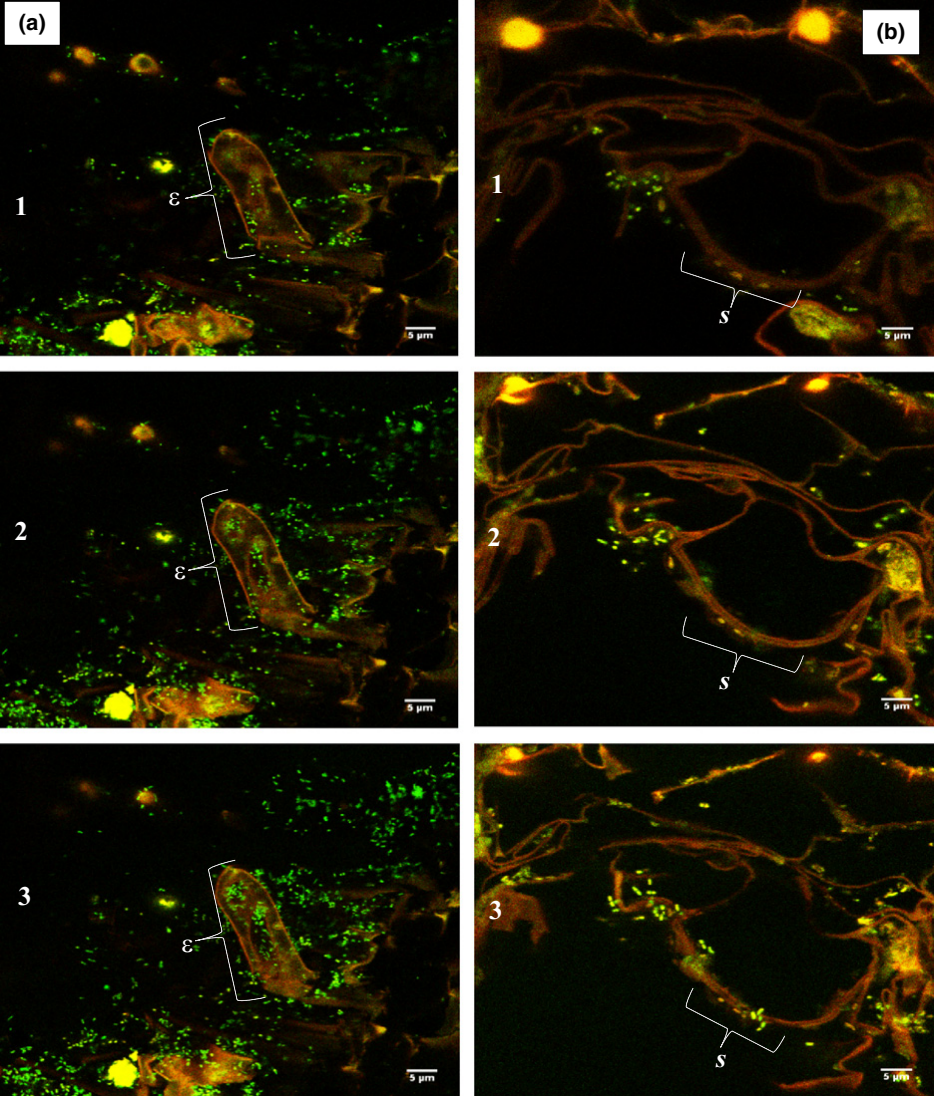
Confocal laser scanning microscopy (CLSM) imaging of (a) *Pseudomonas brassicacearum* and (b) *Rhizobium leguminosarum* colonization of *Brassica juncea* root. ε shows endophytic (interior) colonization and *s* shows rhizospheric (outer sphere) colonization of zinc (Zn) embedded root strand in (a) and (b), respectively. Numbers (1–3) show sequence of image acquisition across the root depth. Green bodies are bacteria cells and orange spots indicate the presence of Zn.

A 3-D reconstruction of CLSM images of un-inoculated *B. juncea* root exposed to zinc contamination shows the absence of bacteria cells (green spots) but some evidence of zinc accumulation (orange spots) (Fig.2a). By contrast, the roots of bacteria inoculated treatments show evidence of bacterial colonization of the roots as well as zinc accumulation (Fig.2b–d). To further understand the relationship between the bacterial strains and zinc accumulation within the root biomass, CLSM images of roots under zinc contamination were acquired at a magnification of ×100 (Fig.3). *P. brassicacearum* exhibited endophytic ability and appeared to colonize the interior of the roots, despite the apparent root accumulation of zinc (see ε in Fig.3a), while *R. leguminosarum* appeared only to be residing in the outer spheres of the root (see *S* in Fig.3b). In all the treatments, the bacterial strains appeared only to colonize the root epidermis with little presence of bacteria cells at the endodermis (see Fig. S1).

Although fluorophore based CLSM analysis was useful in understanding the nature of bacterial colonization and zinc distribution, the spatial relationships between bacteria and zinc could not be deciphered, in part because apart from a few hot spots, the zinc stain was masked by the strong SYTO 9 green fluorescence of the bacteria (Figs 3). Therefore, we conducted a synchrotron based μ-XAS analysis to investigate possible changes in zinc accumulation and speciation due to the direct impacts of bacterial colonization at the root epidermis by comparing these changes with endodermal speciation.

### μ-XAS analysis of zinc distribution and speciation

**Figure 4:**
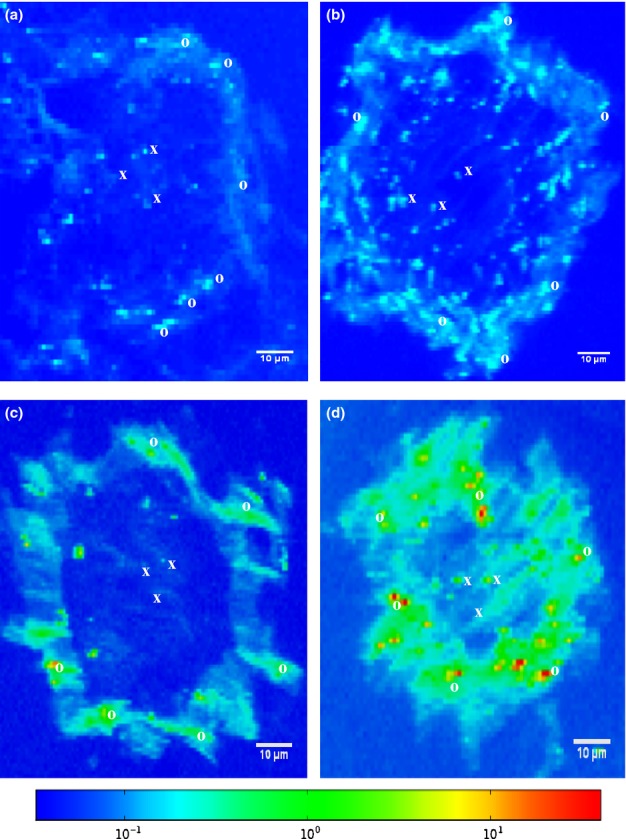
Synchrotron micro X-ray fluorescence (μXRF) imaging of zinc (Zn) in the root of (a) *Brassica juncea* un-inoculated (BZn), (b) inoculated with *Pseudomonas brassicacearum*, (c) *Rhizobium leguminosarum* and (d) combinations of the two bacterial strains, 14 d after seed planting in 400 mg kg^−1^ Zn. Figure shows that the plant growth promoting bacteria (PGPB) significantly enhance Zn sequestration at the epidermis. Symbols o and × represent spots in the root epidermis and endodermis, respectively, that were subjected to microfocus X-ray absorption near edge structure (μXANES) analysis.

Figure4 shows images of synchrotron based μXRF imaging of zinc in plant roots, which are consistent with a higher zinc bioaccumulation in bacteria inoculated treatments compared with the un-inoculated treatment. There were also conspicuous differences in the zinc accumulation pattern between the treatments. In the bacteria inoculated treatments (Fig.4b–d), more of the zinc was localized at the root epidermis in contrast to the un-inoculated treatment (Fig.4a) in which there was a less defined zinc localization pattern at the root epidermis. Moreover, there were noticeable differences between zinc accumulation pattern under *P. brassicacearum* (Fig.4b) and *R. leguminosarum* (Fig.4c). Zinc in the RZn exhibited a characteristic localization pattern at the root epidermis with few high concentration points in the root endodermis, whereas zinc accumulation along the epidermis in the PZn treatments appeared to be more diffuse with more zinc spots in the endodermis than in the RZn treatments. As expected, roots in the combined bacteria (RPZn) treatment combine features of the single bacteria inoculations, resulting in higher zinc accumulation in both the epidermis and endodermis. These zinc bioaccumulation patterns are consistent with our previous observations for *B. juncea* roots grown in standard compost (Adediran *et al*., 2015).

**Figure 5:**
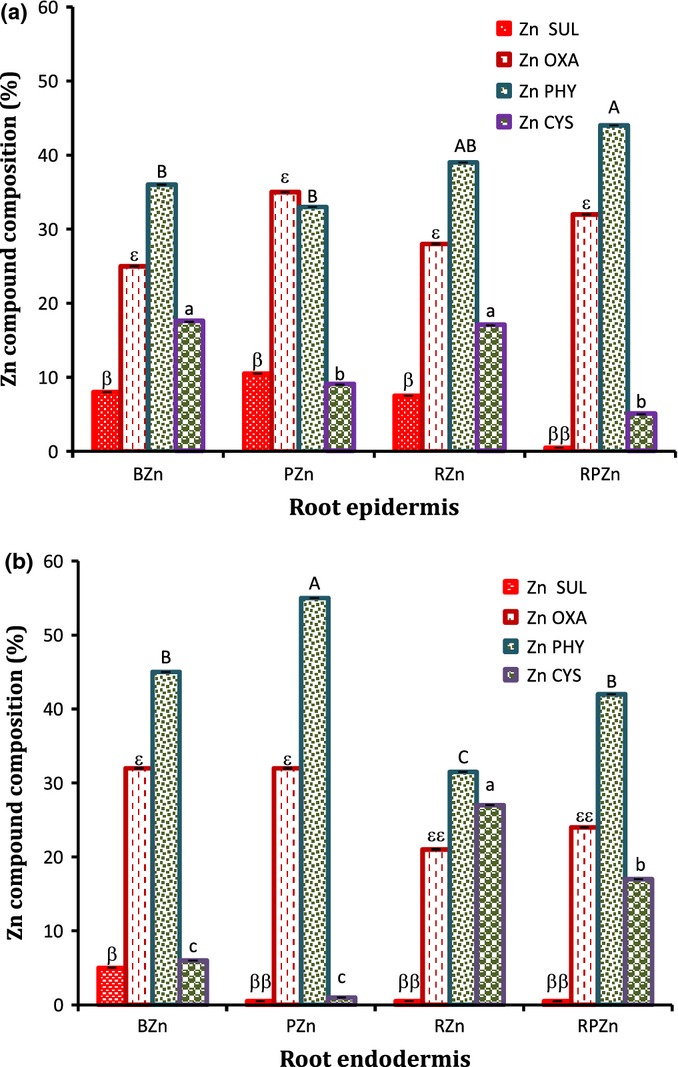
Zinc (Zn) compound compositions (%) in the root (a) epidermis and (b) endodermis of *Brassica juncea*. Zn SUL, zinc sulphate; Zn OXA, zinc oxalate; Zn PHY, zinc phytate; Zn CYS, zinc cysteine. Bars are means of zinc compound compositions from six and three Zn X-ray absorption near edge structure (XANES) collected from the epidermis and endodermis, respectively, of two replicate root samples per treatment. Different alphabets and symbols (upper and lower cases) show significant differences (*P *< 0.05) between treatments.

In the RZn and RPZn treatments in which high zinc bioaccumulation in the seedling root was observed, better root growth was also recorded. This is counter-intuitive, as a higher zinc accumulation in the root biomass should lead to reduced root growth. Possible differences in zinc speciation at the root epidermis and endodermis of the treatments were therefore investigated. Principal component analysis of root μXANES spectra revealed principal zinc binding to sulphate, carbonate, polygalacturonic acid, oxalate, phytate and cysteine-rich ligands. The proportions of these principal zinc components in the μXANES spectra was therefore estimated through LCF and *R* factors, a measure of the goodness of μXANES fittings. Based on physical observation of the zinc K-edge μXANES fits and *R* factor results that range from 0.0004 to 0.0023, the results of LCF were assessed to sufficiently describe zinc speciation in the studied systems (see Figs S2–S5). Although there were no conspicuous differences in the structure of the μXANES for different treatments (see Supporting Information Figs 4), there were significant differences in the proportion of four zinc complexes (sulphate, oxalate, cysteine and phytate) among the treatments (Fig.5).

The same zinc complexes were present in both the epidermis and endodermis of un-inoculated *Brassica juncea* roots. However, there were changes in the zinc speciation between the root epidermis and endodermis of bacterial inoculated plants. In the roots inoculated with *P. brassicacearum* (PZn) and *R. leguminosarum* (RZn), zinc sulphate was only detected at the root epidermis, suggesting minimal sulphate uptake into the root endodermis or that any sulphate taken up is transformed in the endodermis. In the epidermis there were also differences in the proportions of zinc sulphate in the RPZn treatment compared with the others. In the RPZn treatment, zinc sulphate appeared to be completely removed or transformed in the root epidermis and the proportion of zinc present as zinc phytate was higher compared with other treatments.

The percentage zinc oxalate in the root endodermis in the BZn and PZn treatments was significantly higher than in the RZn and the RPZn treatments. The highest percentage of phytate- and cysteine-bound zinc was observed in the endodermis of the PZn and RZn treatments, respectively. The percentage cysteine-bound Zn in RZn and RPZn was significantly higher than in the BZn and PZn treatments, corresponding with a significantly lower percentage of zinc oxalate in the RZn and RPZn treatments.

## Discussion

This study evaluated the ability of *R. leguminosarum* and *P. brassicacearum* to confer zinc tolerance to *B. juncea* and used a combination of CLSM and XAS in an attempt to unravel the mechanisms behind plant growth promotion by these bacteria under zinc toxicity. Although the roots of un-inoculated *B. juncea* and plants inoculated with *P. brassicacearum* were significantly stunted 14 d after exposure to 400 mg kg^−1^ Zn, *R. leguminosarum* significantly promoted plant root growth. Most importantly, inoculation of *B. juncea* with a combination of the two bacteria strains almost completely alleviated the zinc toxicity in plant roots. While *P. brassicacearum* was observed to exhibit endophytic root colonization and significantly to enhance zinc accumulation at both the epidermis and endodermis, *R. leguminosarum* predominantly colonized the root epidermis and significantly enhanced epidermal zinc localization. Analysis of subcellular zinc speciation in roots also revealed the inability of *P. brassicacearum* to enhance root storage of zinc in cysteine-rich complexes/peptides, a less toxic zinc phytochelatin that was significantly synthesized in the endodermis of plants inoculated with *R. leguminosarum*.

The mechanism of plant growth promotion by PGPB has been extensively described by many authors to be based on the bacterial improvement of plant nutrition through nitrogen fixation, phosphate release and siderophores secretion (Khan *et al*., 2009; Compant *et al*., 2010). The apparent absence of any significant improvement in plant growth in uncontaminated treatments, however, suggests that plant nutrients were not a limiting factor and the possibility of plant growth promotion through improvement in plant nutrition was therefore unlikely in this experiment. Moreover, nutrient improvement alone cannot explain the reduced toxicity in the face of higher zinc bioaccumulation. We therefore explored the ability of the PGPB to attenuate zinc toxicity, either through subcellular zinc compartmentalization in the plant root, or through changes in zinc speciation, or through the combination of the two processes as key mechanisms of growth promotion under zinc contamination by PGPB.

### Bacteria–zinc co-localization suggests surface adsorption and cellular uptake in zinc detoxification

Analysis of the CLSM images revealed that the presence of zinc did not prevent bacterial colonization of *B. juncea* roots, as also demonstrated in our previous study (Adediran *et al*., 2015) and also others (Sinha & Mukherjee, 2008; Dimkpa *et al*., 2014). The ability of the bacterial strains to survive zinc contamination has been described by many authors who have identified mechanisms such as efflux of metal ions outside the bacterial cell wall, metallothionein sequestration and reduction of metal ions to less toxic forms (Choudhury & Srivastava, 2001; Miller *et al*., 2009) as possible survival mechanisms. The results from this study further confirm what is known about the colonization patterns of the two bacterial strains. *R. leguminosarum* is a rhizospheric bacterium that mainly resides at the soil–root interface (Reeve *et al*., 2010) and its penetration ability into the root (especially in plants such as *B*. *juncea* that do not have nodules) is expected to be limited compared with *P. brassicacearum* which was isolated from the rhizosplane (a sphere deeper into the root than the rhizosphere) and has been demonstrated to exhibit endophytic properties in this research and in other studies (Long *et al*., 2008). Nevertheless, most of the bacterial cells in *P. brassicacearum* were located in the epidermis (Fig.2b). This may well be driven by the higher root exudates at the root epidermis compared with the root endodermis (el Zahar Haichar *et al*., 2008; Compant *et al*., 2010) providing a labile food source for the bacteria.

Although CLSM could not unambiguously identify zinc localization, the complementary use of μXRF mapping revealed, for the first time the close spatial association between root zinc accumulation and bacterial localization patterns. Specifically, the two PGPB significantly enhanced root zinc bioaccumulation relative to the un-inoculated roots and clearly induced different levels of zinc sequestration at the root epidermis due to the nature of their plant root colonization. The significantly higher zinc tolerance index in roots under *R. leguminosarum* inoculation over *P. brassicacearum* may therefore be due to the isolation of excessive zinc from the endodermis and subsequent zinc deposition at the epidermis where the bacteria was localized. On the other hand, the reduced root tolerance index observed in the PZn treatment may be due to enhanced zinc deposition at the epidermis accompanied by significant zinc transport into the endodermis due to the endophytic root colonization characteristics of *P. brassicacearum*. The RPZn treatments appeared to have benefited from the combination of the rhizospheric and endophytic root colonization nature of both PGPB because the highest amount of zinc accumulation at the root epidermis occurred in this treatment. This spatial association provides circumstantial but strong evidence, for the first time, of the direct physico-chemical mechanisms that PGPB play in metal bioaccumulation and/or detoxification. These direct mechanisms include surface adsorption and subsequent facilitated transport (Kapetas *et al*., 2012) into the endodermis by *P. brassicacearum* and/or cellular uptake and sequestration by both strains. Either mechanism should help to reduce zinc bioavailability to the plant biomass.

### XANES analysis implicates bacteria-induced speciation changes in zinc detoxification

The observation of better root growth in the face of higher zinc accumulation in bacteria inoculated treatments is paradoxical and suggests zinc toxicity attenuation by other mechanisms. Hence, a key element of our study was also to investigate whether changes in speciation were of primary importance in zinc detoxification. Since zinc sulphate solution was the form of zinc contamination used in this experiment it was not surprising that zinc sulphate was detected as a principal zinc component at the root epidermis in all treatments, and in the endodermis of un-inoculated seedlings. The stability constant of ZnSO_4_ in pH conditions similar to the one in plants has been estimated to be log *K *=* *2.38 (Wang *et al*., 1992), suggesting that the compound readily dissociates to yield ionic Zn^2+^ in the plant system. This zinc species offers no protection to the roots and the presence of zinc as zinc sulphate at both root epidermis and endodermis may contribute to the reduced root growth observed in the BZn treatment in this experiment.

The absence of zinc sulphate in bacteria inoculated roots is therefore clear evidence that speciation changes offer a route to zinc detoxification. A significant proportion of zinc exists as zinc oxalate in both root epidermis and endodermis. Complexation of zinc with carboxylic acids such as oxalates and polygalacturonic acids has been reported to be an important response mechanism to metal toxicity in plants under high zinc concentrations (Dalvi & Bhalerao, 2013). Storage of zinc as zinc oxalate has also been observed in hyperaccumulating plants (Sarret *et al*., 2002) and the reaction of zinc with oxalate has been reported to form a more stable zinc oxalate complex (ZnC_2_O_4_) than ZnSO_4_ with a stability constant log *K* = 4.68 (Sillén & Martell, 1964) in plants. However, high concentrations of zinc oxalate complex in metal resistant plants have also been linked with enhanced zinc toxicity to plants (Mathys, 1977; Adediran *et al*., 2015). This may also explain the reduced root growth observed in the BZn and PZn treatments since the proportion of zinc oxalate in their endodermis is significantly higher compared with the endodermis of RZn and RPZn treatments both of which exhibited better root growth. Clearly, enhanced tolerance must be associated with different zinc species than sulphate and/or oxalate complexation.

In our experiment, zinc phytate accounted for the highest proportion of zinc storage in both the epidermis and endodermis in all treatments, except in the epidermis of the PZn treatment where the proportion of zinc oxalate slightly exceeds that of Zn phytate. Immobilization of zinc as stable zinc phytate in plant organs has been observed in other works in both hyperaccumulating and non-hyperaccumulating plants and it is increasingly being recognized as one of the mechanisms for enhanced metal accumulation and tolerance in plant roots (Van Steveninck *et al*., 1990; Kopittke *et al*., 2011). Phytate (myo-inositol hexakis (dihydrogen phosphate), C_6_H_18_O_24_P_6_; IP6) contains strong negatively charged phosphate groups and reacts with zinc to form insoluble zinc phytate complexes in plants (Kopittke *et al*., 2011; Marešová *et al*., 2012). Although globular deposits of zinc phytate are mostly observed in the endodermis of dicotyledonous plants and in the pericycle of monocotyledonous plants, it has also been observed to be deposited in the stele and inner cortex of root epidermis after prolonged exposure to toxic levels of zinc (Van Steveninck *et al*., 1994). Sequestration of zinc as zinc phytate in the root has been reported to restrict root to shoot Zn translocation (Van Steveninck *et al*., 1993). However, zinc sequestration in the form of phytate does not explain the attenuation of zinc toxicity since (1) all treatments, including BZn have high amounts of zinc phytate, suggesting that phytate complexation is not driven by PGPB and (2) the PZn treatments that had the highest proportions of zinc phytate in the plant endodermis exhibited the lowest root tolerance to zinc contamination relative to the RZn and the RPZn treatments.

Finally, we note that in the endodermis of the susceptible BZn and PZn treatments, < 10% of accumulated zinc existed in the form of cysteine-bound complexes, a direct contrast to the RZn and RPZn treatments that showed better zinc tolerance. The better *B. juncea* root tolerance observed under *R. leguminosarum* and its combination with *P. brassicacearum* inoculation in this study is therefore attributed to the bacterial enhanced zinc complexation with cysteine-rich peptides and storage as zinc in these forms in the endodermis of *B. juncea* root.

### Mechanistic model of root tolerance to zinc via bacteria-enhanced synthesis of cysteine-containing peptides

Besides carboxylic acids and phytate, amino acids and peptides are also major cellular ligands known to complex zinc in plants (Salt *et al*., 1999; Zeng *et al*., 2011). Specifically, plants are known to synthesize the peptides phytochelatin from glutathione as a defence mechanism upon exposure to toxic metals. Phytochelatins are synthesized from glutathione under the influence of phytochelatin synthase, as shown by deletion mutants of *Arabidopsis* lacking the phytochelatin synthase gene (Howden *et al*., 1995; Cobbett & Goldsbrough, 2002). Since glutathione is itself synthesized from the amino acids glutamate + cysteine + glycine, the availability of cysteine is a key precursor to its synthesis (Lu, 2013) and hence to that of phytochelatins. Moreover, while the Zn-cysteine observed in this study more likely exists in the form of zinc phytochelatins or glutathione, only the cysteine ligand of these proteins is known to coordinate with zinc (Gelinsky *et al*., 2003; Chekmeneva *et al*., 2008). It is possible therefore that the inferred thiol-based detoxification mechanism is linked to bacteria-enhanced synthesis of cysteine in the presence of *Rhizobium leguminosarum*. Indeed, cysteine has been shown to be important in metal homeostasis, detoxification and tolerance to high concentrations of zinc in many plants (Kelly *et al*., 2002; Zeng *et al*., 2011).

We note that cysteine synthesis in plants is linked to sulphur metabolism (Saito, 2000), whereby sulphur is taken up by plants in its inorganic sulfate form. Accumulated sulphate is then converted to adenosine 5 phosphosulphate (APS) under the influence of the enzyme adenosine triphosphate (ATP) sulphurylase. APS is then enzymatically converted to sulphite by APS reductase and sulphite to sulphide by the ferredoxin-dependent enzyme; sulphite reductase. The combination of sulphite with the carbon skeleton derived from serine (Ser) through O-acetylserine (OAS) synthesis under the influence of Ser acetyltransferase then yields cysteine (Saito, 2000; Tavares *et al*., 2015).

The use of zinc sulphate in our experiments is therefore conducive to cysteine synthesis even in the absence of PGPB, as supported by the detection of cysteine-bound zinc in the epidermis of roots in all four treatments. Indeed, the enzymes ATP sulphurylase and APS reductase have been reported to be secreted in *B. juncea* upon exposure to metals (Heiss *et al*., 1999). However, the observation that more cysteine-bound zinc was present in the endodermis of roots with *R*. *leguminosarum* leads us to propose additional induction of cysteine synthesis by this bacterium as the main mechanism for zinc detoxification and enhanced root accumulation under *R*. *leguminosarum* inoculation. Such a mechanism is supported in part by studies showing that the gene sequence of *Rhizobium leguminosarum* includes acetyl transferases *lacA* and *cysE* genes (Downie, 1989) for secreting Ser acetyltransferase (Hindson *et al*., 2000; Parker *et al*., 2001). The genome of *Rhizobium leguminosarum* bv.* trifolii* strain WSM1325 used in our experiment has been fully characterized (Reeve *et al*., 2010) and comprehensive information about its gene sequence, Ser O-acetyltransferase secretion, sulphur metabolism and amino acid biosynthesis has been organized into an online database available at http://www.genome.jp/dbget-bin/www_bget?rlg:Rleg_3685.

**Figure 6:**
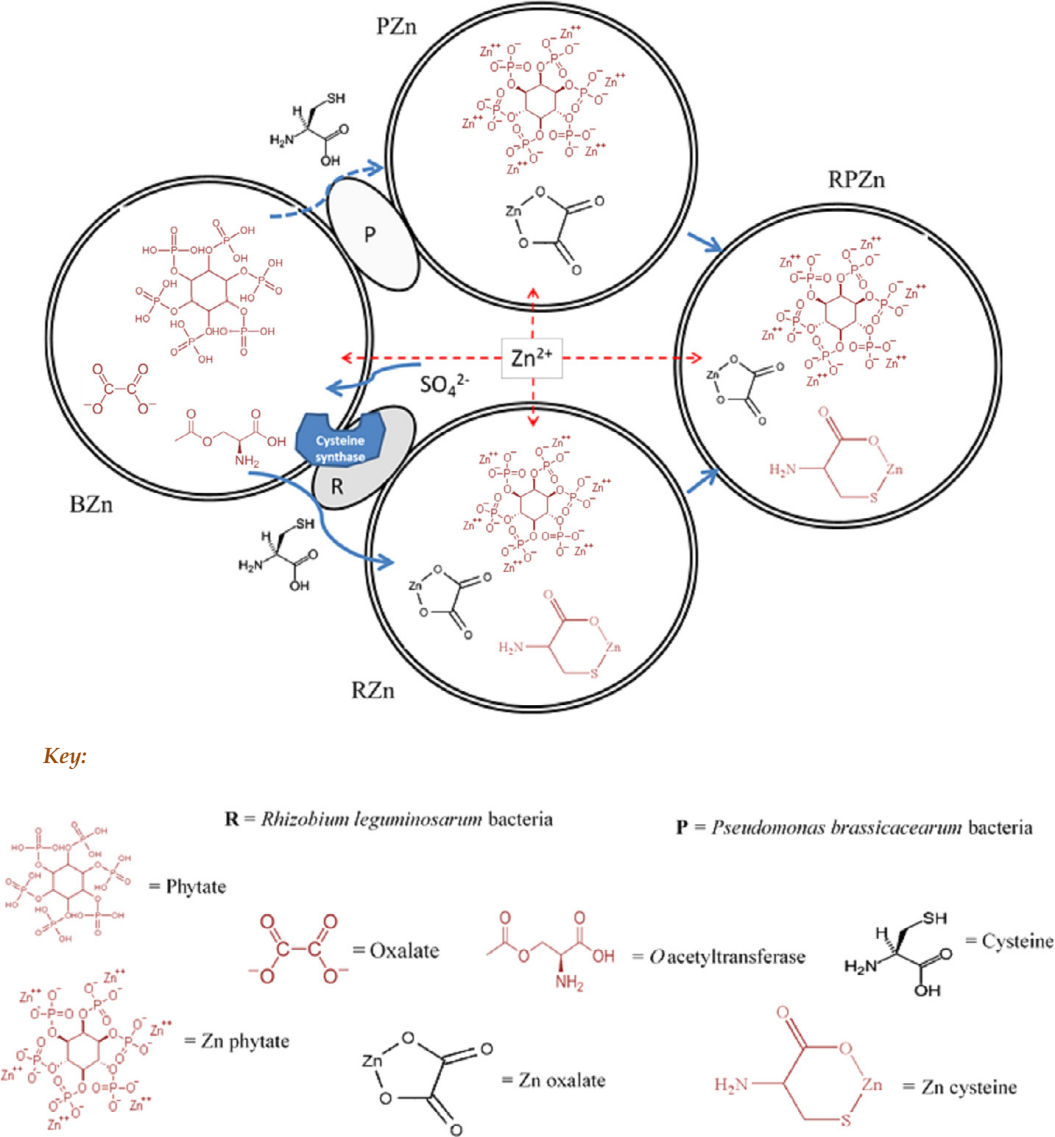
Schematic model of zinc (Zn) speciation and detoxification mechanisms deduced from this research for *Brassica juncea* roots (BZn) inoculated with *Rhizobium leguminosarum* (RZn), *Pseudomonas brassicacearum* (PZn) and a combination of the two bacterial strains (RPZn) upon exposure to 400 mg kg^−1^ Zn contamination as zinc sulphate. Figure depicts the native presence of phytate, oxalate and *O-*acetylserine, with uptake of sulphate enhancing cysteine synthesis from O-acetylserine in BZn roots. Although all three ligands are capable of zinc complexation, induction of additional cysteine synthesis by *R. leguminosarum* in RZn increases cysteine concentration and increases endodermal zinc chelation as cysteine bound peptide compared with PZn, whereas the combination of the two bacteria in RPZn leads to a more balanced distribution between phytate and cysteine complexation in root endodermis. Better root growth was observed in RZn and RPZn which correlated with higher proportion of cysteine bound zinc in root endodermis compared with BZn and PZn treatments.

Figure6 is a schematic representation of our mechanistic analysis, taking into account bacterial localization based on CLSM, zinc distribution from MicroXRF mapping and the preceding speciation modelling results. The scheme assumes the presence in both epidermal and endodermal tissue of oxalate, phytate and O-acetylserine, whence the ingress of sulphate leads to the synthesis of cysteine from O-acetylserine. All three ligands are then capable of complexing zinc in proportions that depend on the concentration of the ligand and the relative stability constants of the complexes. Thus, at low cysteine concentrations, such as is apparently the case in BZn and PZn, most zinc is complexed by phytate in the endodermis, owing to its higher stability constant of log *K c*. 11 at low ionic strengths (Cigala *et al*., 2010) relative to log *K* = 9.80 for the Zn–cysteine complex (Marešová *et al*., 2012). Induction of additional cysteine synthesis by *R. leguminosarum* increases the cysteine concentration and increases the proportion of cysteine-bound zinc, whereas the combination of the two bacteria leads to a more balanced distribution between phytate and cysteine complexation in the endodermis, possibly reflecting the endophytic localization of *P. brassicacearum* to enhance Zn accumulation and the detoxifying properties of cysteine complexation by *R. leguminosarum*. Apparently, the high stability constant of Zn-cysteine also offers a higher resistance to protolytic degradation compared with the Zn organic acid complexes and therefore helps to reduce the cellular bioavailability of Zn (II) (Cobbett & Goldsbrough, 2002; Kelly *et al*., 2002).

In conclusion, apart from the enhanced epidermal zinc sequestration in roots inoculated with *R. leguminosarum*, enhanced cysteine synthesis and subsequent endodermal zinc sequestration by cysteine-containing phytochelatins in the endodermis of RZn and RPZn treatments was identified to be responsible for the enhanced root tolerance to zinc observed. Although *P. brassicacearum*; the endophytic PGPB of *Brassica* appeared to prevent zinc sulphate accumulation in the endodermis of roots exposed to zinc sulphate, its inability to mediate accumulation of zinc as zinc oxalate and especially favour the sequestration of zinc in cysteine-rich peptides in endodermis, is responsible for the observed stunted root growth in PZn.

We therefore propose enhanced epidermal zinc sequestration and bacterial mediated reduction in zinc toxicity through synthesis of cysteine-rich ligands in root endodermis, as key complementary mechanisms of plant growth promotion in plants inoculated PGPB such as *R. leguminosarum* under zinc sulphate contamination. Our findings have direct relevance to PGPB enhanced phytoremediation of soils contaminated with zinc mining waste, since most zinc comes from zinc sulphides, and in soils contaminated with emerging engineered nanomaterials such as ZnS. In both cases, zinc bioavailability is driven by oxidative dissolution (often driven by bacteria) of the sulphide to sulphate that acts as a source of sulphate for cysteine synthesis. It remains to be demonstrated therefore, whether a different mechanism would operate if zinc was supplied in forms other than sulphate.

## Supporting Information

**Fig. S1** 3-D reconstruction of root (a) epidermis and (b) endodermis in plant inoculated with *P. brassicacearum* and *R. leguminosarum*, and exposed to zinc for 14 d. **Fig. S2** Zn K-edge XANES fitting, R-factors and Zn-compound compositions for epidermis and endodermis of un-inoculated root (BZn). **Fig. S3** Zn K-edge XANES fitting, R-factors and Zn-compound compositions for epidermis and endodermis of root inoculated with *P. brassicacearum* (PZn). **Fig. S4** Zn K-edge XANES fitting, R-factors and Zn-compound compositions for epidermis and endodermis of root inoculated with *R*. *leguminosarum* (RZn). **Fig. S5** Zn K-edge XANES fittings, R-factors and Zn-compound compositions for epidermis and endodermis of root inoculated with a combination of *R. leguminosarum* and *P. brassicacearum* (RPZn). **Table S1** Freshly prepared or purchased Zn standards used for XANES analysis
