# Supplementary material for: Bacteria–zinc co-localization implicates enhanced synthesis of cysteine-rich peptides in zinc detoxification when Brassica juncea is inoculated with Rhizobium leguminosarum
Source: New Phytol. 2015 Aug 11;209(1):280–93. doi: 10.1111/nph.13588 (PMC4676334; doi:10.1111/nph.13588)
Supplement: Supplementary file 1 [file nph0209-0280-sd1.pdf]

New Phytologist

Bacteria–zinc co-localization implicates enhanced synthesis of cysteine-rich peptides in zinc detoxification when *Brassica juncea* is inoculated with *Rhizobium leguminosarum*

Gbotemi A. Adediran, Bryne T. Ngwenya, J. Frederick W. Mosselmans and Kate V. Heal

Article acceptance date: 8 July 2015

The following Supporting Information is available for this article: Table S1 and Figs S1–S5

Table S1 Freshly prepared or purchased Zn standards used for XANES analysis

Zn standard	Characteristics
Zn oxalate	7.0 mM $Zn(NO_3)_2$ + 70 mM sodium oxalate, pH 7.0
Zn phosphate	7.0 mM $Zn(NO_3)_2$ + 70 mM sodium phosphate, pH 7.0
Zn histidine	7.0 mM $Zn(NO_3)_2$ + 80 mM histidine, pH 7.0
Zn cysteine	7.0 mM $Zn(NO_3)_2$ + 70 mM cysteine, pH 7.0
Zn phytate	7.0 mM $Zn(NO_3)_2$ + 70 mM phytic acid solution, pH 7.0
Zn polygalacturonate	7.0 mM $Zn(NO_3)_2$ + 70 mM polygalacturonic acid solution, pH 7.0
Zn formate	7.0 mM $Zn(NO_3)_2$ + 70 mM formic acid solution, pH 7.0
Zn sulfate, Zn nitrate, Zn citrate, Zn acetate and Zn carbonate	Purchased from Sigma Aldrich

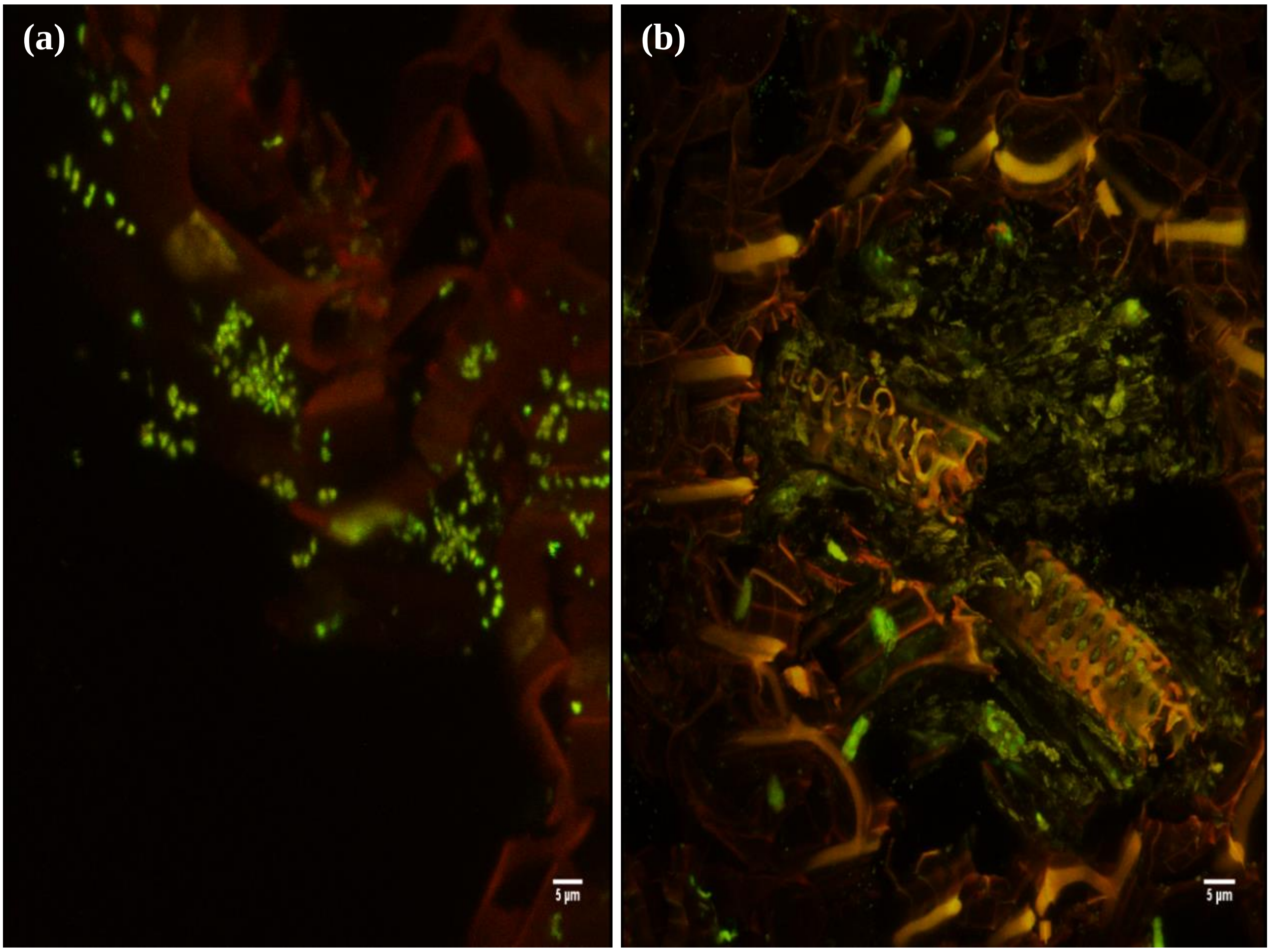

Fig. S1 3-D reconstruction of root (a) epidermis and (b) endodermis in plant inoculated with ***P. brassicacearum*** **and** ***R. leguminosarum*****, and exposed to Zn for 14 d. Regular green bodies (< 5 μm) are bacteria cells and the red colour indicates Zn distribution. (a) Shows bacteria colonisation at the root epidermis while (b) shows the endodermis relatively free of bacterial cells (note: the green fluorescent bodies in (b) are from the DNA of plant cells not bacterial cells and it is not possible to distinguish between the strains since they both appear green.**

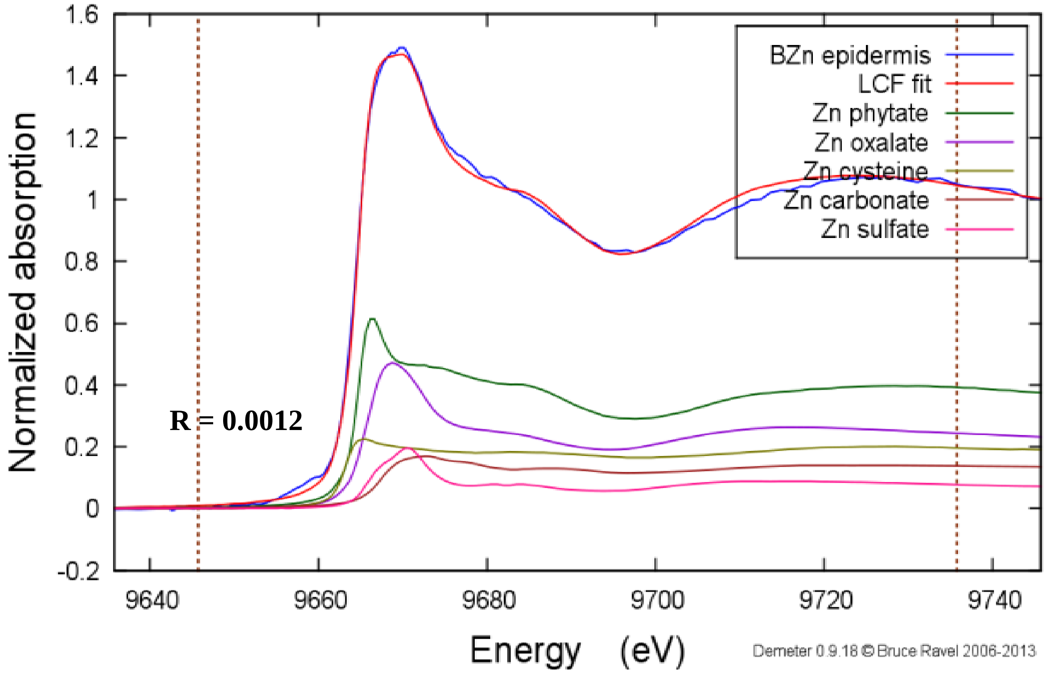

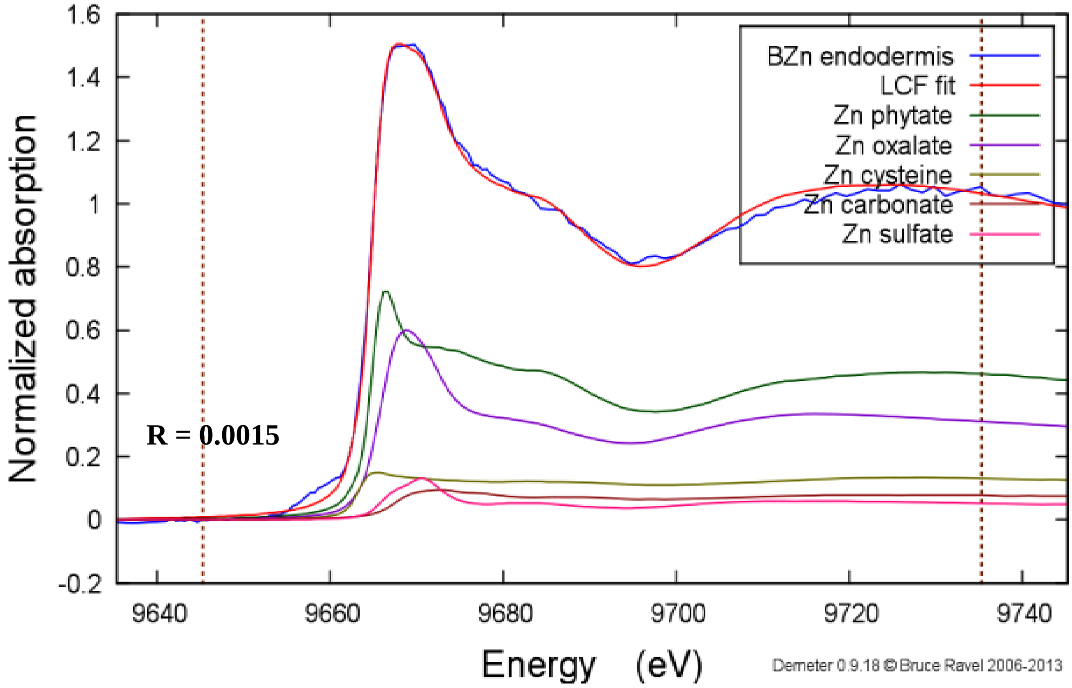

Fig. S2 Zn K-edge XANES fitting, R-factors and Zn-compound compositions for epidermis and endodermis of un-inoculated root (BZn). ***R*** **= Σi (experimental-fit)^2 / Σi (experimental)^2. The lower the** ***R*** **value the better the fit.**

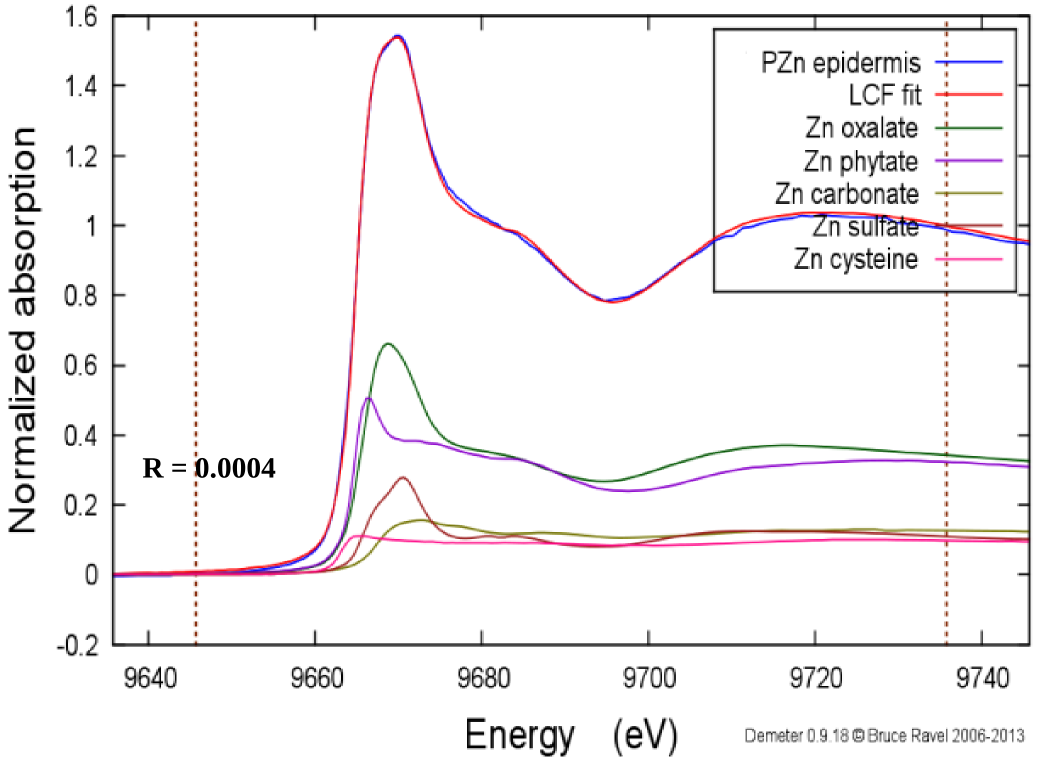

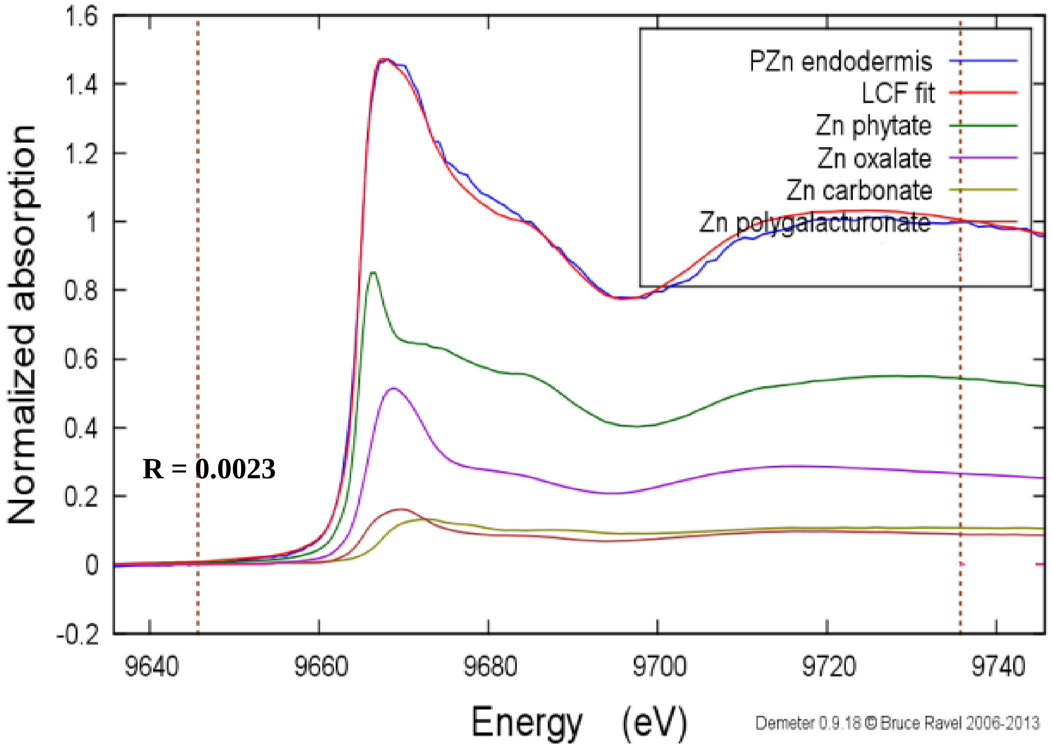

Fig. S3 Zn K-edge XANES fitting, R-factors and Zn-compound compositions for epidermis and endodermis of root inoculated with *P. brassicacearum* (PZn). R = Σi (experimental-fit)^2 / Σi (experimental)^2. The lower the *R* value the better the fit.

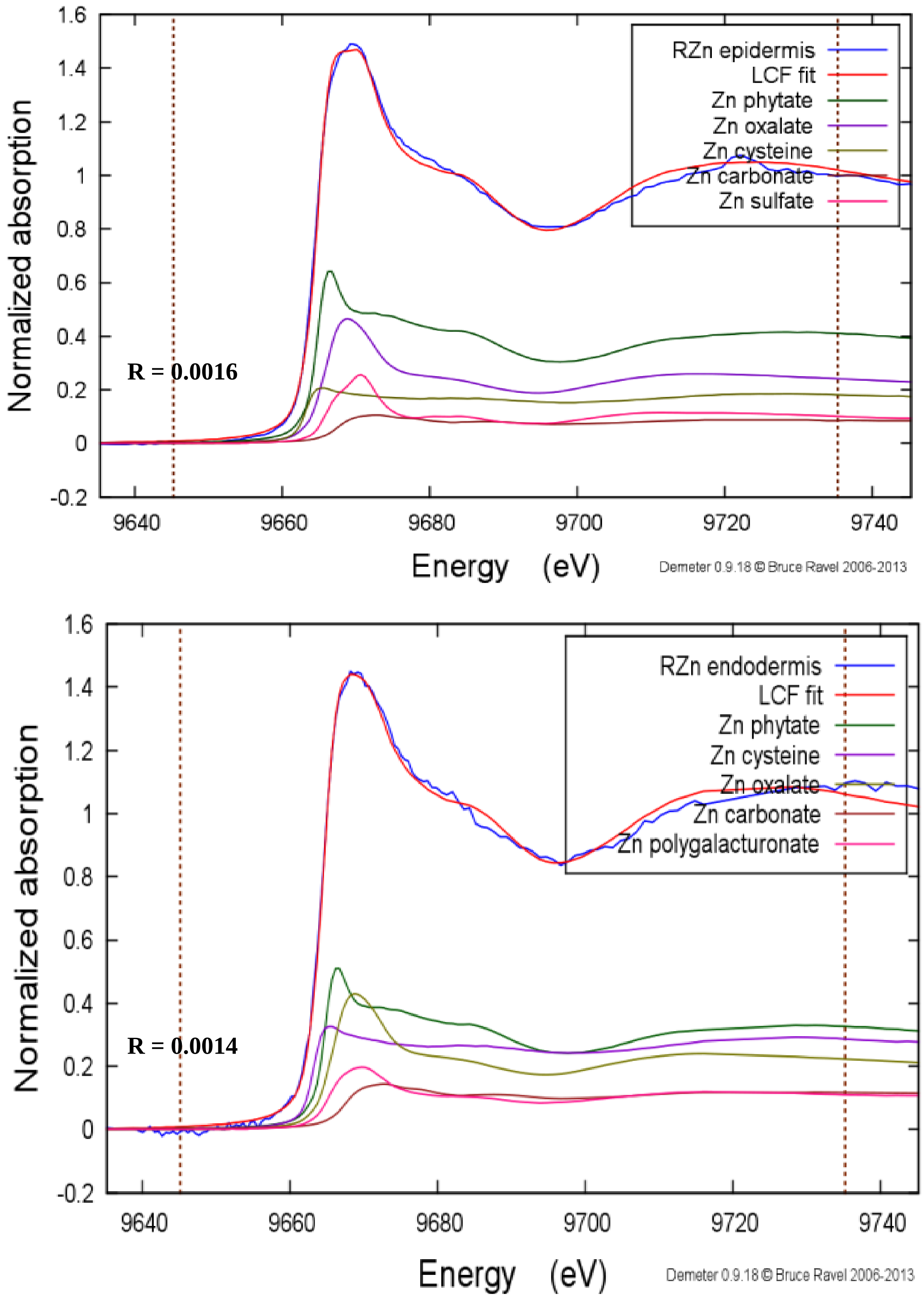

Fig. S4 Zn K-edge XANES fitting, R-factors and Zn-compound compositions for epidermis and endodermis of root inoculated with *R. leguminosarum* (RZn). $R = \Sigma i\ (\text{experimental-fit})^2 / \Sigma i\ (\text{experimental})^2$. The lower the *R* value the better the fit.

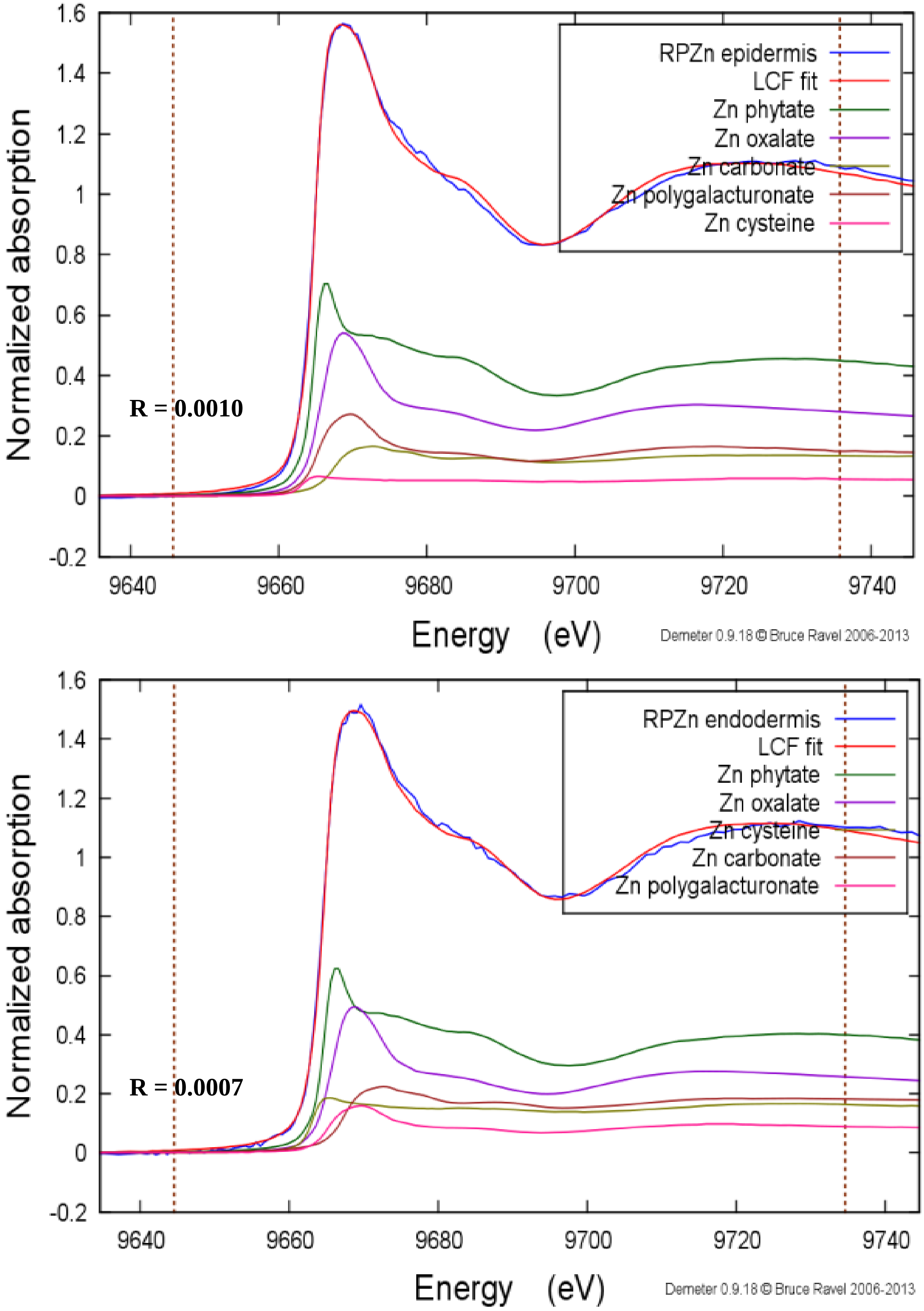

Fig. S5 Zn K-edge XANES fittings, R-factors and Zn-compound compositions for epidermis and endodermis of root inoculated with a combination of *R. leguminosarum* and *P. brassicacearum* (RPZn). $R = \Sigma i\ (\text{experimental-fit})^2 / \Sigma i\ (\text{experimental})^2$. The lower the *R* value the better the fit.
